# Prevalence and Prognostication of CD5+ Mature T-Cell Lymphomas

**DOI:** 10.3390/cancers16193430

**Published:** 2024-10-09

**Authors:** Omar Elghawy, Miao Cao, Jason Xu, Daniel J. Landsburg, Jakub Svoboda, Sunita D. Nasta, Elise A. Chong, Stephen J. Schuster, Colin J. Thomas, Jordan S. Carter, Montreh Tavakkoli, Marco Ruella, Stefan K. Barta

**Affiliations:** 1 Department of Medicine, University of Pennsylvania, Philadelphia, PA 19104, USA; omar.elghawy@pennmedicine.upenn.edu (O.E.); miao.cao@students.jefferson.edu (M.C.); jason.xu@pennmedicine.upenn.edu (J.X.); montrehtavakkoli@gmail.com (M.T.); 2 Thomas Jefferson University Department of Pharmacology, Physiology and Cancer Biology, Philadelphia, PA 19107, USA; 3 Lymphoma Program, Abramson Cancer Center, University of Pennsylvania, Philadelphia, PA 19104, USA; daniel.landsburg@pennmedicine.upenn.edu (D.J.L.); jakub.svoboda@pennmedicine.upenn.edu (J.S.); sunita.nasta@pennmedicine.upenn.edu (S.D.N.); elise.chong@pennmedicine.upenn.edu (E.A.C.); stephen.schuster@pennmedicine.upenn.edu (S.J.S.); colin.thomas1@pennmedicine.upenn.edu (C.J.T.); jordan.carter@pennmedicine.upenn.edu (J.S.C.); mruella@upenn.edu (M.R.)

**Keywords:** T-cell lymphoma, prognostication, outcomes, CD5, cellular markers, flow cytometry, ATLL, PTCL

## Abstract

**Simple Summary:**

T-cell lymphomas are hematologic cancers that are difficult to manage due to their aggressive nature and poor response to traditional chemotherapies. Response rates are unpredictable even in the era of novel therapeutics, and there has yet to be a clinically validated cellular marker associated with response rates or treatment outcomes. CD5 is a cellular marker that has been associated with poor outcomes in other cancers but has not been well established in T-cell lymphoma. This study confirmed that CD5 has a high prevalence in T-cell lymphomas and is associated with poor prognostic markers such as advanced age, bone marrow involvement, and poor functional status. Additionally, CD5 was associated with inferior survival in T-follicular cell lymphoma and adult T-cell lymphoma. These results suggest that CD5 is associated with worse prognosis in T-cell lymphomas and may be a potential therapeutic target in future studies.

**Abstract:**

**Background:** T-cell lymphomas (TCLs) are a group of heterogenous cancers with poor rates and duration of response. There remains a great challenge in risk stratification of these cancers. Cluster of differentiation (CD) 5 has shown prognostic implication in many subtypes of B-cell lymphoma; however, its role in TCLs is not known. **Methods:** We performed a single-institution retrospective analysis of newly diagnosed patients with TCL. CD5 positivity was determined based on positive results via immunohistochemistry and/or flow cytometry. We used univariate and multivariable analysis of biological factors to assess their association with survival outcomes. **Results:** A total of 194 patients with TCL spanning 14 subtypes were identified. CD5 positivity was noted in 63% of patients, with the highest proportion of CD5 expression in TFH TCL (93.9%), PTCL-NOS (82.9%), and ATLL (77.8%) (*p* = 0.00004). Older age at diagnosis (*p* = 0.001), stage III or IV disease (*p* = 0.05), and bone marrow involvement (*p* = 0.003) were also associated with CD5 expression. Complete response rates were numerically lower in patients with CD5+ TCL across all subtypes. OS/PFS was not statistically associated with CD5 status in the overall cohort; however there was significantly decreased OS in CD5+ TFH TCL (*p* = 0.04) and CD5+ ATLL (*p* = 0.04) patients. **Conclusions:** This study represents the first to examine CD5 expression as a prognostic biomarker for outcomes in TCL. The frequent expression of CD5 in the most common nodal TCL in the Western world underpins its potential as an attractive target for cellular therapies. Confirmation of these findings in a larger cohort and investigation of potential pathophysiological mechanisms explaining our observations are planned.

## 1. Introduction

Mature T-cell lymphomas (TCLs) are a group of rare, aggressive, and heterogenous non-Hodgkin lymphomas (NHLs) which comprise only 6–10% of NHLs in the United States [1]. The median time from initial diagnosis to disease relapse or progression after initial treatment for TCL is 6.7 months, with secondary median overall survival (OS) and median progression-free survival (PFS) of 5.5. and 3.1 months, respectively [2]. The 5th edition of the World Health Organization (WHO) classification of hematolymphoid tumors defines 30 distinct subtypes of TCL [3]. The subtypes of TCL are classified based on cell of origin, clinic presentation, cytomorphology, and molecular genetics with various clinical outcomes [4]. The rare and heterogeneous nature of TCL as a disease group poses a challenge in risk stratification, and thus, reliable prognostic markers are desired to guide patient care.

**Figure 1:**
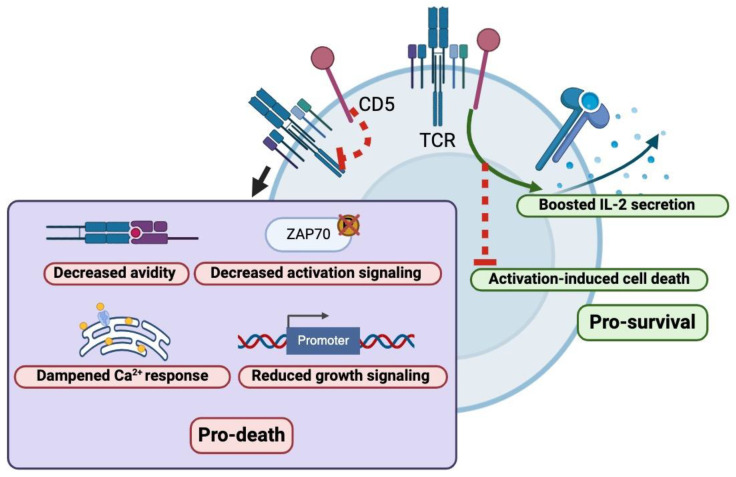
Intracellular impact of activation and inhibition of CD5 within T-lymphocytes. Figure created using BioRender.com.

Cluster of differentiation (CD) 5 is a transmembrane glycoprotein expressed on the surface of thymocytes, T lymphocytes, and a small subset of B- lymphocytes [5] and has canonically been used as a T-cell marker in clinical practice. CD5 is composed of three Scavenger Receptor Cysteine-Rich (SRCR) domains and has been a well-described regulator of T-cell proliferation [6] (Figure 1). CD5 functions by activating at least three well-described biological pathways that are crucial for cell survival, metabolism, and immune function. Firstly, CD5 augments the MAPK (Ras/Erk) pathway, which is crucial in the amplification and integration of cellular signals to induce physiologic responses including cellular differentiation, proliferation and regulation of apoptosis [7]. Secondly, CD5 amplifies the Ca^2+^–calmodulin–calcineurin–NFAT pathway via TRPC1, which regulates the activity of transcription factors of activated T-cells and plays a crucial role in adaptive immunity [8]. Lastly, CD5 activation has been shown to modulate the PI3-K/Akt/mTOR pathway via the p85 unit of PI3-K, which has been a well-described mediator of growth, proliferation, survival, and metabolism [9]. Derangements of any of these pathways have been well described in the pathogenesis of various cancers and have been molecular targets for potential therapies for decades but have proven difficult to safely inhibit [10]. Interestingly, preclinical studies of intracellular pathways prompted by CD5 were shown to be similar to those of anergic B cells and B-CLL cells [11].

CD5 has also been shown to be implicated in other biological pathways involved in T-cell proliferation and regulation; however, the exact mechanisms are less clear. For instance, CD5 has been shown to serve as a co-stimulatory receptor for T-cell receptor (TCR) activation by providing necessary secondary stimulatory signals and promoting interleukin-2 production [12,13]. Other studies, however, have also revealed CD5 as a negative mediator for TCR signal transduction through several mediators, including SHP1, CBL, and CBL-B to tune down TCR activation, and translocation into the TCR immunological synapse upon phosphorylation [14,15]. It appears that the impact of CD5 on TCR signal transduction appears to be context-dependent based on cellular location (thymus vs. periphery) and based on the strength of TCR stimulation; however, the nuances of this phenomenon are an active area of scientific research [16]. Given that CD5 is expressed on all T-cell subtypes, CD-5 is also inherently involved in modulation of the immune environment and relative ratios between Treg/effector cells [17]. The in vivo administration of CD5 antibodies has been shown to enhance the generation of induced Treg (iTreg) cells [18]. It is therefore speculated that the inhibition of CD5 would simultaneously reduce iTreg cell number and activate effector functions on conventional T-cells, thereby increasing T-cell reactivity against self and potentiating auto-immune disorders. CD5 has also been shown to upregulate Th17-cell differentiation via the modulation of cellular Interferon-γ response and retinoic acid receptor-related orphan receptor-γt localization, although the exact mechanism by which this occurs is unknown [19].

Studies of CD5 in some subsets of B-cell lymphomas, such as follicular lymphoma, diffuse large B-cell lymphoma, and mantle cell lymphoma, have demonstrated that the expression of CD5 is associated with a worse clinical outcome [20,21,22], whereas loss of CD5 expression is associated with better survival [23]. CD5 in TCL, however, has not been extensively studied despite its crucial role in T-cell proliferation and regulation. This study aims to characterize the expression patterns of CD5 in different subsets of TCL and evaluate whether CD5 expression has a prognostic role in these cancers.

## 2. Materials and Methods

A single-institution retrospective analysis of patients treated for a histologic diagnosis of T-cell lymphoma from 1 January 2017 to 1 January 2023 at the University of Pennsylvania was performed. Patients were identified through a query of the University of Pennsylvania/Abramson Cancer Center database. Baseline demographics, disease characteristics, treatment history, toxicities, and clinical outcomes were abstracted from the electronic medical record in accordance with the University of Pennsylvania Institutional Review Board and the Declaration of Helsinki. We defined CD5 positivity (≥1%) based on positive results via immunohistochemistry or flow cytometry in available tumor tissue. A flow cytometry gating strategy was utilized where cells were gated based on their forward scatter and side scatter. Duplicate and dead cells were excluded, and CD3+ cell were gated followed by CD4- and CD8- cell gating. CD5 on each CD4+/CD8+ T-cells was gated 30% from the right as high expression (bright) and 30% from the left as low expression (dim). Disease response was determined based on the documented clinician assessment in the electronic medical record.

All statistical tests were conducted using R v4.3.2. Baseline characteristics were assessed via independent *t*-test, Fisher’s exact test, and 2-sided Pearson chi-square analysis, as appropriate. A survival analysis was conducted using the Kaplan Meier methodology with log rank testing and Cox proportional hazard models. Overall survival (OS) and progression free survival (PFS) were co-primary end points, measured from time of treatment to death (OS) and/or progression (PFS), respectively, and censored at date of last follow-up. A univariate analysis of the patient and disease-specific characteristics, including functional status, age, stage, TCL type and extranodal/bone marrow involvement was performed, and their association with CD5 expression was assessed, as well as their association with respect to survival. Next, we performed a multivariable analysis of biological factors associated with survival outcomes on a univariate analysis according to PFS and/or OS. A *p*-value of <0.05 was considered significant.

In order to contextualize our single-center data, we obtained T-ALL Cellular Indexing of Transcriptomes and Epitopes by sequencing (CITE-seq) data from T-cell developmental reference from Tan et al. [24]. CITE-seq data were normalized using CLR normalization across cells (margin = 2). Original cell annotations and dimensional reductions were retained. The surface and RNA expression for CD2 and other T-cell markers were plotted in *n* = 16,199 healthy thymocytes using the VlnPlot function in Seurat v5. All code generated for processing of the CITE-seq are available at https://github.com/tanlabcode/SC_TALL (accessed on 3 March 2024).

## 3. Results

### 3.1. Patient and Tumor Characteristics

We identified 194 patients with newly diagnosed T-cell lymphoma spanning 14 subtypes. The patient information is reported in Table 1. The median age at diagnosis was 61 years (range: 19–85), and 55% of patients were male. Most patients identified as White (70.1%), followed by African American (19.7%). At presentation, 54% harbored B-symptoms and 23% of patients had ECOG status ≥2. The most common TCL subtypes were T-follicular helper (TFH) TCL (25%), followed by peripheral T-cell lymphoma not otherwise specified (PTCL-NOS) (21%) and anaplastic lymphoma kinase (ALK) negative anaplastic large cell lymphoma (ALCL) (14%). Sixty-three percent of patients had extranodal sites involved at presentation (including stage IE and non-nodal TCL subtypes), and 47% had stage IV disease (Table 2). A total of 23% of patients had bone marrow involvement, and 18% had bulky disease (tumors > 7.5cm) at diagnosis (Table 2).

**Figure 2:**
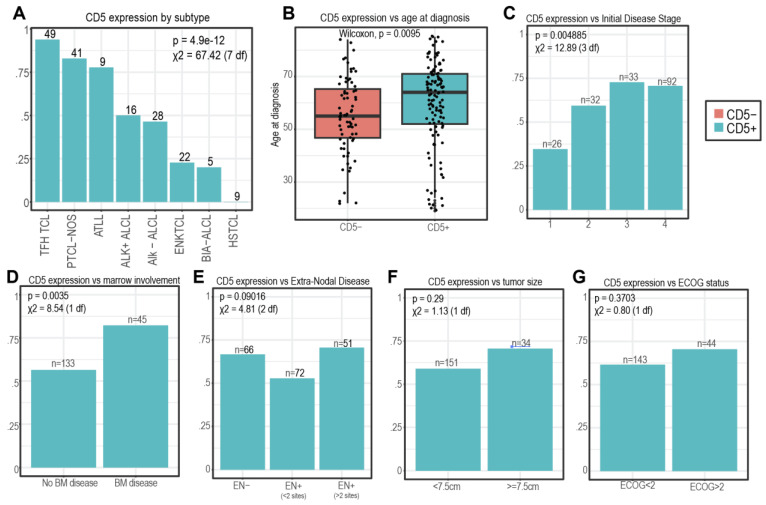
**CD5 positivity in n = 194 patients of T-cell lymphoma**. The proportion of CD5+ is shown (**A**) in 8 subtypes with n > 5 patients, (**B**) vs. age at diagnosis, (**C**) by initial disease stage, (**D**,**E**) by marrow and extra-nodal involvement, (**F**) by initial tumor size, and (**G**) by ECOG status. In panels (**A**,**C**–**G**), the *p*-values are shown above the chi-square test statistic. In panel (**B**), the *p*-value from the 2-sided Wilcoxon test is shown.

CD5 positivity was noted in 63% of patients. Eighty-seven percent of CD5+ patients (106) had positive CD5 on IHC. Most patients (104, 85%) with CD5 positivity had CD5+ flow cytometry. Of this subset, 81 (78%) had bright CD5, while the remainder had dim CD5 (22%). The proportion of CD5+-expressing tumors was significantly different across different subtypes of TCL, with T-follicular helper TCL (TFH TCL, 93.9%), peripheral T-cell lymphoma not otherwise specified (PTCL-NOS, 82.9%), and adult T-cell leukemia/lymphoma (ATLL, 77.8%) having the highest proportion of CD5+ patients (Figure 2A, *p* = 0.00004). In contrast, extranodal NK TCL (ENK TCL, 22.7%), breast implant-associated ALCL (BIA ALCL, 20%), and hepatosplenic TCL (HSTCL, 0%) had the lowest CD5 expression.

### 3.2. CD5 Expression and Patient/Disease Characteristics

On the univariate analysis, we found that older age at diagnosis was associated with CD5 expression, with the median age of CD5+ patients being 64 (range: 19–85) compared to 55 (range: 22–84) for CD5- patients (*p* = 0.001, Figure 2B). CD5 expression was more common in advanced stage disease (stage III/IV: 71.2%) compared to stage I/II disease (48.2%, *p* = 0.0045, Figure 2C) and in patients with bone marrow involvement (82.2% vs. 56.4%, *p* = 0.003, Figure 2D). We did not detect statistically significant associations between CD5 positivity and extranodal disease (*p* = 0.09), tumor size (*p* = 0.29), or ECOG status (*p* = 0.37) at presentation (Figure 2E–G).

We then performed a multivariable analysis using logistic regression models where CD5 positivity was used as a binary responding variable and modeled against sex, subtype, stage at diagnosis, race, ECOG status, B-symptoms, and extranodal disease. This analysis also revealed statistically significant association of CD5 positivity with the PTCL-NOS and TFH TCL subtypes (*p* = 0.014, *p* = 0.02, Supplementary Table S1). In addition, several variables associated with poor prognosis were noted to have robust associations at the edge of statistical significance, including the ATLL and ALK+ ALCL subtypes (*p* = 0.1, *p* = 0.15), ECOG performance status >2 (*p* = 0.055), and presence of B-symptoms (*p* = 0.13). We did not observe statistically significant associations between CD5 positivity and sex, stage at diagnosis, extranodal disease, or age at diagnosis (Supplementary Table S1).

### 3.3. CD5 Status and Outcomes

**Figure 3:**
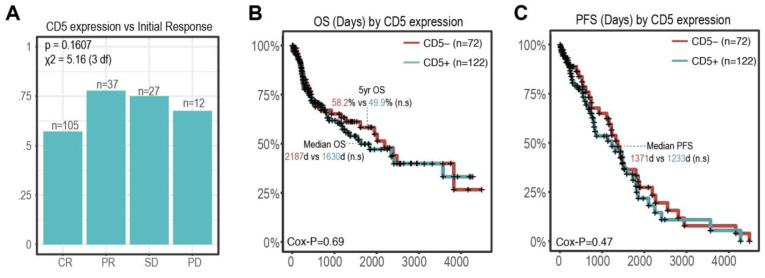
**CD5 status and response to treatment.** (**A**) Proportion of CD5+ patients by initial response. *p*-values are shown above the chi-square test statistic with 3 degrees of freedom. (**B**,**C**) OS and PFS Kaplan Meier curves by CD5 expression status. Log likelihood *p*-value from Cox proportional hazard test is shown in the bottom left. Key survival summary statistics (5-Yr OS, median OS/PFS) are labeled within each graph. (n.s: non significant).

The outcomes for the entire patient cohort are shown in Table 3. Patients who had complete responses to initial therapy had the lowest proportion of CD5+ patients (57.1%; n = 60/105), while those with partial responses (67.6%; n = 25/37), stable disease (77/8%; n= 21/27), and progressive disease (75%; n = 9/12) had a higher proportion of CD5+ patients; this trend was however not statistically significant (*p* = 0.16, Figure 3A). There was a positive trend towards improved outcome in CD5- patients; the 5-year survival for CD5- patients (n = 72) was 58.2% (95 CI: 46.7–72.6%) compared to 49.9% (95 CI: 39.8–62.5%) in CD5 patients (Figure 3B). The median OS (73 months vs. 54 months) and PFS (45.7 months vs. 41.1 months) were both longer in CD5- patients (*p* = 0.69 and *p* = 0.47, respectively, Figure 3B,C); however, this also did not reach the predefined statistical significance (Figure 3B,C).

**Figure 4:**
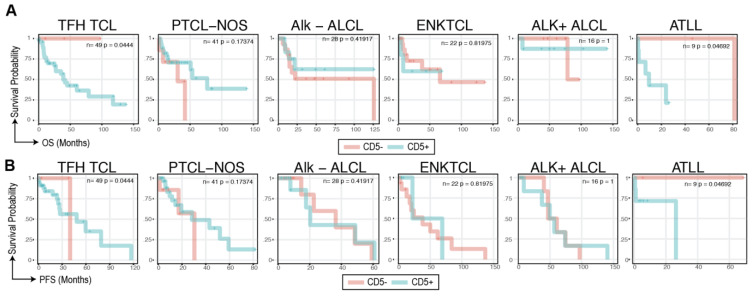
**Subtype-specific survival analyses reveal CD5+ association with poor prognosis in TFH TCL and ATLL.** (**A**) OS Kaplan Meier curves for subtypes with >5 patients stratified by CD5 expression status. The log likelihood *p*-value from the Cox proportional hazard test is shown in the top right. (**B**) PFS Kaplan Meier curves for subtypes with >5 patients stratified by CD5 expression status.

We next performed a subtype-specific survival analysis. Of our 194 patients, 6 subtypes had >5 patients and could be stratified into CD5+ and CD5- arms for survival analyses. We found CD5 status to be associated with overall (Figure 4A) and progression free survival (Figure 4B) in TFH TCL (n = 49, *p* =0.04) and ATLL (n = 9, *p* = 0.04), where the CD5-positivity was associated with worse prognosis. In TFH TCL, the median OS in CD5+ patients were 39.6 months (95% CI: 27.5 months—NR) and was not reached in CD5- patients. In ATLL, the median OS for CD5+ patients was 9.7 months (95% CI: 0.93 months—15.2 months) vs. 82.7 months (56.7 months-103.3 months) in the CD5- arm. CD5 status in other subtypes, including PTCL-NOS (n = 41), ALK-mutated/wt ALCL (ALK-mut: n = 16, WT: n = 28), and ENKTCL (n = 22) did not reach statistical significance (Figure 4A,B).

**Figure 5:**
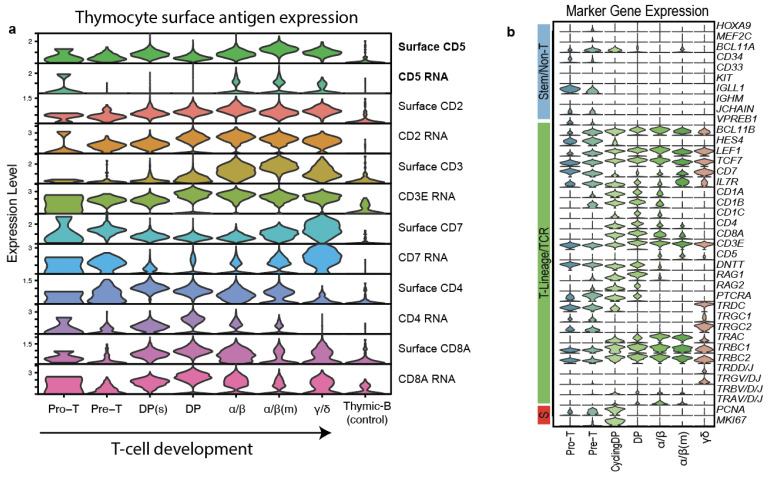
Surface and mRNA expression of selected surface antigens in developing thymocytes. (**a**) Antibody-derived-tag (ADT) surface expression and mRNA expression for CD2, CD3, CD5, CD7, and CD4/8 from n = 16,119 thymocytes profiled by CITE-seq are plotted. Cell types are organized in order of T-cell development; thymic B-cells are shown as a control. Cycling DP are represented by “DP(s)”, and mature α/β (CCR7+, CCR9-) are indicated by “α/β(m)”. (**b**) RNA gene expression supporting cell annotations. Markers are grouped by lineage and cell cycle.

Finally, to contextualize the findings from our single-center experience and to investigate the feasibility of CD5 as a potential therapeutic target in TCL, we explored CD5 expression across T-cell development using previously published T-cell developmental references profiled using Cellular Indexing of Transcriptomes and Epitopes by sequencing (CITE-Seq, 10 × 3′ scRNA-seq + antibody-derived tags). This analysis revealed low but detectable surface CD5 at the Pro-T (CD34+ CD1A-) stage, rising expression in the Pre-T (CD34+ CD1A+) and double positive cell states (CD4/8+), and peak expression in functional alpha-beta T-cells (CD4/8 single-positive, PTCRA-, TRAV/D/J+, Figure 5a,b). Notably, across all stages of T-cell development, we detected the expression of surface CD5 despite low or minimal RNA expression, in line with the higher sensitivity of surface-level staining compared to 3′ mRNA capture and the persistence of stable surface proteins compared to mRNA (Figure 5a). These findings were in line with the differences in CD5 positivity across TCL subtypes based on their putative cell of origin.

## 4. Discussion

To our knowledge, this study represents the first to examine CD5 expression as a prognostic biomarker in TCL. In our cohort, CD5 expression was common across most TCL types, with CD5 positivity highest in the two most common nodal TCL subtypes TFH/AITL and PTCL-NOS. CD5 was more frequently observed in patients with poor prognostic markers, such as advanced age, higher stage, and bone marrow involvement. Complete response rates were numerically lower in the CD5+ cohort; however, this was not statistically significant. While progression and overall survival were not statistically associated with CD5 status in the overall cohort, they were significantly inferior in TFH TCL and ATLL in patients expressing CD5. Using CITE-sequencing, we detected robust expression of surface CD5 on immature and mature T-cell subsets. Notably, surface CD5 expression was robust even in T-cells with low or minimal CD5 mRNA expression.

Similar to the results of this study, the impact of CD5 positivity in B-cell NHL is largely dependent on the subtype of lymphoma. In contrast to TCLs, the expression of CD5 in B-cell neoplasms is far less common and classically indicative of chronic lymphocytic leukemia (CLL) or mantle cell lymphoma (MCL) [25]. CLL, a usually indolent disease, is unique in that CD5 positivity has been shown to be associated with superior outcomes to CD5- cases with a longer time to treatment (TTT) of 13.0 years in the CD5+ group compared to 5.8 years in the CD5- group and lower rates of lymphadenopathy (31.5% vs. 51.4%) and splenomegaly (16.1% vs. 42.1%) [26]. Conversely, CD5- MCL patients have been shown to have a more favorable prognosis than CD5+ MCL patients with a significantly longer progression-free survival (PFS) and a tendency for longer overall survival independently of other favorable prognostic markers such as IGHV hypermutation, absence of SOX11 expression, low Ki-67, and κ light chain restriction [27].

The rate of incidence of CD5 positivity is drastically decreased across the other subtypes of B-cell NHLs; however, the role of CD5 as a prognostic marker in these cancers has been extensively explored. Similarly to MCL, CD5+ follicular lymphoma (FL) and CD5+ marginal zone lymphoma (MZL) have also been shown to be associated with worse outcomes compared to their CD5- counterparts. In retrospective studies, CD5+ FL exhibited significantly shorter mPFS (44 months vs. 89 months) and mOS (154 months vs. 222 months) [28]. CD5+FL has also been associated with the expression of CD25 and MUM1, a leukemic phase, a lesser frequency of t (14;18) (q32; q21), a higher IPI, and a higher rate of transformation into DLBCL [29,30,31]. Likewise, CD5+ MZL has been shown to have higher rates of transformation to diffuse large B-cell lymphoma and have lower response rates (30% vs. 77%), PFS (25% vs. 45% at 3 years), and OS (44% vs. 77%) to rituximab compared to their CD5- counterparts [32]. A recent retrospective study has also demonstrated that while CD5 expression was not associated with survival outcomes, CD5+ MZL patients were found to have a higher incidence of bone marrow involvement, similar to our findings [33].

Despite its low frequency in all variants of diffuse large B-cell lymphoma (DLBCL), CD5(+) DLBCL is the most well-studied CD5+ B-cell neoplasm with poorer outcomes compared to CD5- DLBCL [34]. CD5+ DLBCL is characterized by drastically worse PFS/OS and a high frequency of central nervous system relapse after standard immunochemotherapy [35]. A recent study investigated whether more intense therapies may improve outcomes in CD5+ DLBCL. The PEARL5 (a Phase II trial of DA-EPOCH and Rituximab with high dose-MTX therapy for newly diagnosed DLBCL with CD5 expression) study demonstrated the efficacy of the DA-EPOCH-R with a high-dose methotrexate regimen for CD5+ DLBCL and for the first time demonstrated improved ORR between CD5+ and CD5- DLBCL (80.0% vs. 63.8% respectively) although further studies are identifying additional regiments to treat these cancers [36].

Over the past decade, our understanding of TCL pathobiology has grown significantly and while no universally accepted gold standard exists for the treatment of patients with newly diagnosed or relapsed TCLs, the treatment paradigms for these cancers has drastically evolved in recent years. Therapies for TCL based on cytotoxic chemotherapy remain suboptimal, with 34% of patients experiencing primary refractory disease and the majority of patients relapsing within three years of treatment [26,27]. Therefore, there has been a push to develop novel therapies with several new treatments including immunotherapy. Targets with clinically useful agents in TCL include CD52, CCR4, and CD25 [37,38,39,40,41]. Enhancing our understanding of surface marker expression and their role in the pathobiology therefore are essential.

The next frontier in immunotherapies in TCL is cellular therapy, specifically chimeric antigen receptor T-cell (CART) therapy, which utilizes engineered T-cells to eliminate malignancies. The use of FDA-approved CD19-directed CART therapies has greatly improved the survival and outcomes of advanced stage B cell malignancies. CD5 has been explored as a CART therapy target for TCL in preclinical and clinical settings [42]. Therefore, this study is highly relevant in establishing the frequency and role of CD5 in TCL [43]. Targeting CD5 via CART is promising, as seen in the MAGENTA study and recent Phase I studies of donor-derived CD5 CAR T-cells in patients with relapsed or refractory T-cell acute lymphoblastic leukemia [44,45].

We were able to demonstrate that CD5 expression is dynamic during T-cell development and expression-frequency-dependent on the putative cell of origin of the respective T-cell malignancy. Given its high expression in TCLs, CD5 holds significant potential as a therapeutic target, particularly in nodal mature T-cell lymphomas. CD5 has been shown to be integral to the native antitumor response as a negative regulator of human dendritic cells and tumor infiltrating lymphocytes [46]. A great deal of work has also gone into the utilization of the anti-tumor effect of CD5-targeting fully human heavy-chain variable (FHVH) domains, which bind directly to different epitopes of the CD5 antigen [47].

Additionally, several preclinical studies have shown that the genetic deletion of CD5 in CAR and TCR T-cells enhances expansion, persistence, and cytotoxicity in both solid tumor and hematological malignancy models [48,49]. These results suggest that CD5 may be a critical negative regulator of CAR T-cells that can be therapeutically targeted. In vivo, deleting CD5 has led to increased expansion, persistence, and activity of CAR T-cells or NK CAR cells [50]. Furthermore, the deletion of CD5 in this setting allows for a reduction in fratricide in CART-cell manufacture, thereby making it an enticing treatment option for the use of CD5+ TCLs.

## 5. Conclusions

In our study, over 75% of patients with TFH-TCL, PTCL-NOS, and ATLL were CD5+ at the initial presentation, justifying that these CD5-directed adoptive cell therapies have the potential to significantly improve the outcomes of TCL patients. This study also suggests that CD5 may be a possible prognostic indicator for TCLs, which is relevant as to date, few definitive biological biomarkers that reliably predict treatment outcome have been identified in TCL.

Despite this, our study has several limitations. Firstly, the inherent nature of the rarity and heterogeneity of TCL restricted our cohort size, posing challenges to studying its prognostic and predictive potential for disease outcomes. Due to our small sample size, we were unable to meaningfully stratify patients by potential co-founding factors such as initial treatment, response to initial treatment, and cytogenetic and mutational status. Moreover, as a retrospective study, no standardized follow-ups nor scanning frequency was established, which may lead to inconsistency in data collection. We were also unable to assess the strength of expression of CD5 and its implications on outcome, although CD5 is usually strongly and uniformly expressed on malignant T-cells. Therefore, exploring its role in a larger multi-institution dataset is being planned. Ultimately, a better understanding of the biological roles of CD5 in various lineages of normal T-cells and subtypes of malignant T-cells will also help to elucidate the clinical significance of CD5 expression and is highly relevant, with CD5 as an attractive target for cellular therapies in TCL.

## Figures and Tables

**Table 1:** Patient demographics/disease subtypes of the study cohort.

Characteristic	Value (%)	CD5+	CD5−
	*n =* 194	*n = 122*	*n = 72*
**Sex**			
Male	107 (55.2%)	59	48
Female	87 (44.8%)	63	24
**Race**			
Caucasian	136 (70.1%)	83	53
African American	37 (19.1%)	25	12
Asian	7 (3.6%)	4	3
Hispanic/Latino	6 (3.1%)	5	1
Other	1 (0.5%)	1	0
Unknown	7 (3.6%)	4	3
**ECOG**			
<2	143 (73.7%)	88	55
≥2	44 (22.7%)	31	13
Unknown	7 (3.6%)	3	4
**Stage**			
I	26 (13.4%)	9	17
II	32 (16.4%)	19	13
III	33 (17%)	24	9
IV	92 (47%)	65	27
Unknown	11 (5.6%)	5	6
**B-Symptoms at presentation**			
Yes	105 (54.1%)	70	35
No	82 (42.3%)	46	36
Unknown	7 (3.6%)	6	1
**Subtype**			
T-follicular-helper (TFH) TCL (including angioimmunoblastic T-cell lymphoma [AITL])	49 (25.3%)	46	3
Peripheral T-cell lymphoma not otherwise specified (PTCL-NOS)	41 (21.1%)	34	7
Anaplastic lymphoma kinase negative anaplastic large cell lymphoma (ALK- ALCL)	28 (14.4%)	13	15
Extranodal NK/T-cell lymphoma (ENK TCL)	22 (11.3%)	5	17
Anaplastic lymphoma kinase positive anaplastic large cell lymphoma (ALK+ ALCL)	16 (8.2%)	8	8
Adult T-cell leukemia/lymphoma (ATLL)	9 (4.6%)	7	2
Hepatosplenic T-cell lymphoma (HSTCL)	9 (4.6%)	0	9
Breast implant-associated lymphoma (BIA-ALCL)	5 (2.6%)	1	4
Subcutaneous panniculitis-like T-cell lymphoma (SPTCL)	4 (2.1%)	2	2
Primary cutaneous gamma-delta T-cell lymphoma (PCGD-TCL)	3 (1.5%)	2	1
Enteropathy-associated T-cell lymphoma disease (EATL)	3 (1.5%)	2	2
Monomorphic epitheliotropic intestinal T-cell lymphoma (MEITL)	2 (1%)	0	2
Primary cutaneous CD30-positive T-cell lymphoproliferative disorder (CD30+ CTCL/LPD)	1 (0.5%)	1	0
T-cell prolymphocytic leukemia (T-PLL)	1 (0.5%)	1	0

**Table 2:** Disease characteristics of the study cohort.

Characteristic	Value (%)	CD5+	CD5−
Bulky disease			
Yes	34 (17.5%)	24	10
No	151 (77.8%)	89	62
Unknown	9 (4.6%)	9	0
**LDH**			
≥ULN	106 (54.6%)	74	32
<ULN	60 (30.9%)	34	26
Unknown	28 (12.9%)	14	14
**CD5 status**			
Positive *	122 (62.9%)		
CD5 > Dim	103 (53.1%)		
Negative	72 (37.1%)		
**B-symptoms at presentation**			
Yes	105 (54.1%)	70	35
No	82 (42.3%)	46	36
Unknown	7 (3.6%)	6	1
**Extra-nodal disease**			
Yes	123 (63.4%)	74	49
>2 sites	51 (26.3%)	36	15
No	66 (34%)	44	22
Unknown	5 (2.5%)	4	1
**Bone marrow disease**			
Yes	45 (23.2%)	37	8
No	133 (68.6%)	75	58
Unknown	16 (8.2%)	10	6

* Defined as any malignant CD5+ cells on flow or CD5-dim IHC.

**Table 3:** Response to treatment in the patient cohort.

Characteristic	Value (%)	CD5+	CD5−
Initial Response			
CR	105 (54.1%)	60	45
PR	27 (13.9%)	21	6
SD	12 (6.2%)	9	3
PD	37 (19.1%)	25	12
Unknown	13 (0.7%)	4	3
**Time of Progression**			
None	65 (33.5%)	35	30
Refractory *	73 (37.6%)	58	20
Relapse/Progression	42 (21.6%)	21	16
Unknown	5 (2.6%)	3	2
**Alive at Last Follow-Up**			
Alive	111 (57.2%)	71	40
Deceased	83 (42.8%)	51	32
**Survival Outcomes**			
**1-year OS**	154 (76.3%)	96	58
**2-year OS**	137 (66.4%)	87	50
**Cause of death**			
Lymphoma	49 (25.3%)	33	16
Treatment related	17 (8.8%)	5	7
Within 30 days	12 (6.2%)	1	6
>30 days	5 (2.6%)	4	1
Other	4 (2.1%)	3	1
Unknown	118 (60.8%)	73	45

* Defined as partial response or stable disease that did not fully respond to therapy but did not exhibit progression of disease.

## Data Availability

The original contributions presented in the study are included in the article/Supplementary Materials; further inquiries can be directed to the corresponding author/s.

## Supplementary Materials

The following supporting information can be downloaded at https://www.mdpi.com/article/10.3390/cancers16193430/s1, Table S1: Multivariable modeling of diagnostic variables on CD5.

## References

[1] Marchi E., O’Connor O.A. (2020). The rapidly changing landscape in mature T-cell lymphoma (MTCL) biology and management. CA A Cancer J. Clin..

[2] Mak V., Hamm J., Chhanabhai M., Shenkier T., Klasa R., Sehn L.H., Villa D., Gascoyne R.D., Connors J.M., Savage K.J. (2013). Survival of patients with peripheral T-cell lymphoma after first relapse or progression: Spectrum of disease and Rare Long-Term Survivors. J. Clin. Oncol..

[3] Li W., Li W. (2022). The 5th Edition of the World Health Organization Classification of Hematolymphoid Tumors. Leukemia.

[4] Alaggio R., Amador C., Anagnostopoulos I., Attygalle A.D., de Oliveira Araujo O., Berti E., Bhagat G., Borges A.M., Boyer D., Calaminici M. (2022). The 5th edition of the World Health Organization Classification of Haematolymphoid Tumours: Lymphoid Neoplasms. Leukemia.

[5] Tarakhovsky A., Kanner S.B., Hombach J., Ledbetter J.A., Müller W., Killeen N., Rajewsky K. (1995). A role for CD5 in TCR-mediated signal transduction and thymocyte selection. Science.

[6] Soldevila G., Raman C., Lozano F. (2011). The immunomodulatory properties of the CD5 lymphocyte receptor in health and disease. Curr. Opin. Immunol..

[7] Garaud S., Taher T.E., Debant M., Burgos M., Melayah S., Berthou C., Parikh K., Pers J.-O., Luque-Paz D., Chiocchia G. (2018). CD5 expression promotes IL-10 production through activation of the MAPK/Erk pathway and upregulation of TRPC1 channels in B lymphocytes. Cell. Mol. Immunol..

[8] Freitas C.M.T., Johnson D.K., Weber K.S. (2018). T Cell Calcium Signaling Regulation by the Co-Receptor CD5. Int. J. Mol. Sci..

[9] Glaviano A., Foo A.S.C., Lam H.Y., Yap K.C.H., Jacot W., Jones R.H., Eng H., Nair M.G., Makvandi P., Geoerger B. (2023). PI3K/AKT/mTOR signaling transduction pathway and targeted therapies in cancer. Mol. Cancer.

[10] Sanchez-Vega F., Mina M., Armenia J., Chatila W.K., Luna A., La K.C., Dimitriadoy S., Liu D.L., Kantheti H.S., Saghafinia S. (2018). Oncogenic Signaling Pathways in The Cancer Genome Atlas. Cell.

[11] Taher T.E., Bystrom J., Mignen O., Pers J.O., Renaudineau Y., Mageed R.A. (2020). CD5 and B lymphocyte responses: Multifaceted effects through multitudes of pathways and channels. Cell. Mol. Immunol..

[12] Ceuppens J.L., Baroja M.L. (1986). Monoclonal antibodies to the CD5 antigen can provide the necessary second signal for activation of isolated resting T cells by solid-phase-bound OKT3. J. Immunol..

[13] Vandenberghe P., Ceuppens J.L. (1991). Immobilized anti-CD5 together with prolonged activation of protein kinase C induce interleukin 2-dependent T cell growth: Evidence for signal transduction through CD5. Eur. J. Immunol..

[14] Voisinne G., García-Blesa A., Chaoui K., Fiore F., Bergot E., Girard L., Malissen M., Burlet-Schiltz O., Gonzalez de Peredo A., Malissen B. (2016). Co-recruitment analysis of the CBL and CBLB signalosomes in primary T cells identifies CD5 as a key regulator of TCR-induced ubiquitylation. Mol. Syst. Biol..

[15] Brossard C., Semichon M., Trautmann A., Bismuth G. (2003). CD5 inhibits signaling at the immunological synapse without impairing its formation. J. Immunol..

[16] Peña-Rossi C., Zuckerman L.A., Strong J., Kwan J., Ferris W., Chan S., Tarakhovsky A., Beyers A.D., Killeen N. (1999). Negative regulation of CD4 lineage development and responses by CD5. J. Immunol..

[17] Tabbekh M., Mokrani-Hammani M., Bismuth G., Mami-Chouaib F. (2013). T-cell modulatory properties of CD5 and its role in antitumor immune responses. Oncoimmunology.

[18] Voisinne G., Gonzalez de Peredo A., Roncagalli R. (2018). CD5, an Undercover Regulator of TCR Signaling. Front. Immunol..

[19] McGuire D.J., Rowse A.L., Li H., Peng B.J., Sestero C.M., Cashman K.S., De Sarno P., Raman C. (2014). CD5 enhances Th17-cell differentiation by regulating IFN-γ response and RORγt localization. Eur. J. Immunol..

[20] Durani U., Ansell S.M. (2021). CD5+ diffuse large B-cell lymphoma: A narrative review. Leuk Lymphoma.

[21] Xu Y., Sun W., Li F. (2020). De Novo CD5+ Diffuse Large B-Cell Lymphoma: Biology, Mechanism, and Treatment Advances. Clin. Lymphoma Myeloma Leuk..

[22] Cabeçadas J., Nava V.E., Ascensao J.L., Gomes da Silva M. (2021). How to Diagnose and Treat CD5-Positive Lymphomas Involving the Spleen. Curr. Oncol..

[23] Tan K., Xu J., Chen C., Vincent T., Pölönen P., Hu J., Yoshimura S., Yu W., Sussman J., Chen C.H. (2023). Identification and targeting of treatment resistant progenitor populations in T-cell Acute Lymphoblastic Leukemia. Res. Sq..

[24] Demir C., Kara E., Ekinci Ö., Ebinç S. (2017). Clinical and Laboratory Features of CD5-Negative Chronic Lymphocytic Leukemia. Med. Sci. Monit..

[25] Friedman D.R., Guadalupe E., Volkheimer A., Moore J.O., Brice Weinberg J. (2018). Clinical outcomes in chronic lymphocytic leukaemia associated with expression of CD5, a negative regulator of B-cell receptor signalling. Br. J. Haematol..

[26] Miao Y., Lin P., Saksena A., Xu J., Wang M., Romaguera J., Yin C.C., Medeiros L.J., Li S. (2019). CD5-negative Mantle Cell Lymphoma: Clinicopathologic Correlations and Outcome in 58 Patients. Am. J. Surg. Pathol..

[27] Li Y., Hu S., Zuo Z., Hong M., Lin P., Li S., Konoplev S., Wang Z., Khoury J.D., Young K.H. (2015). CD5-positive follicular lymphoma: Clinicopathologic correlations and outcome in 88 cases. Mod. Pathol..

[28] Miyoshi H., Sato K., Yoshida M., Kimura Y., Kiyasu J., Ichikawa A., Ishibashi Y., Arakawa F., Nakamura Y., Nakashima S. (2014). CD5-positive follicular lymphoma characterized by CD25, MUM1, low frequency of t (14; 18) and poor prognosis. Pathol. Int..

[29] Mayson E., Saverimuttu J., Cartwright K. (2014). CD5-positive follicular lymphoma: Prognostic significance of this aberrant marker?. Intern. Med. J..

[30] Sekiguchi Y., Imai H., Wakabayashi M., Sawada T., Ichikawa K., Komatsu N., Noguchi M. (2011). CD5-positive follicular lymphoma: A case report and literature review. Intern. Med..

[31] Xia Y., Ge J., Sun Z., Nan F., Wan W., Xu D., Zhang M., Fu X. (2022). CD5-positive marginal zone lymphoma: Clinicopathological features and survival outcomes. Leuk. Res..

[32] Ghione P., Bantilan K.S., Joffe E., Palomba M.L., Noy A., Caron P., Hamlin P., Kumar A., Matasar M., Owens C. (2024). CD5 expression in marginal zone lymphoma does not predict inferior outcome and has similarities to indolent lymphomas. Blood Neoplasia.

[33] Ma D., Ma Y., Ma Y., Liu J., Gu Y., Liu N., Xiang C., Liu H., Sang W. (2022). Molecular subtyping of CD5+ diffuse large B-cell lymphoma based on DNA-targeted sequencing and Lymph2Cx. Front. Oncol..

[34] Nato Y., Miyazaki K., Maruyama D., Takahashi H., Sunami K., Murakami S., Negoro E., Miyazawa Y., Choi I., Okada T. (2023). Survival and CNS Relapse in Patients with CD5-Positive Diffuse Large B-Cell Lymphoma: A Multi-Institutional Observational Study in Japan. Blood.

[35] Zhang F., Li L., Zhang L., Li X., Fu X., Wang X., Wu J., Sun Z., Kong F., Ren L. (2019). Prognostic analysis of CD5 expression in double-hit diffuse large B-cell lymphoma and effectiveness comparison in patients treated with dose-adjusted EPOCH plus rituximab/R-CHOP regimens. Blood Lymphat. Cancer.

[36] Reimer P., Rüdiger T., Geissinger E., Weissinger F., Nerl C., Schmitz N., Engert A., Einsele H., Müller-Hermelink H.K., Wilhelm M. (2009). Autologous Stem-Cell Transplantation as First-Line Therapy in Peripheral T-Cell Lymphomas: Results of a Prospective Multicenter Study. JCO.

[37] Kim Y.H., Bagot M., Pinter-Brown L., Rook A.H., Porcu P., Horwitz S.M., Whittaker S., Tokura Y., Vermeer M., Zinzani P.L. (2018). Mogamulizumab versus vorinostat in previously treated cutaneous T-cell lymphoma (MAVORIC): An international, open-label, randomised, controlled phase 3 trial. Lancet Oncol..

[38] Horwitz S., O’Connor O.A., Pro B., Illidge T., Fanale M., Advani R., Bartlett N.L., Christensen J.H., Morschhauser F., Domingo-Domenech E. (2019). Brentuximab vedotin with chemotherapy for CD30-positive peripheral T-cell lymphoma (ECHELON-2): A global, double-blind, randomised, phase 3 trial. Lancet.

[39] Horwitz S.M., Scarisbrick J.J., Dummer R., Whittaker S., Duvic M., Kim Y.H., Quaglino P., Zinzani P.L., Bechter O., Eradat H. (2021). Randomized phase 3 ALCANZA study of brentuximab vedotin vs physician’s choice in cutaneous T-cell lymphoma: Final data. Blood Adv..

[40] Dearden C.E., Khot A., Else M., Hamblin M., Grand E., Roy A., Hewamana S., Matutes E., Catovsky D. (2011). Alemtuzumab therapy in T-cell prolymphocytic leukemia: Comparing efficacy in a series treated intravenously and a study piloting the subcutaneous route. Blood.

[41] Foss F.M., Zinzani P.L., Vose J.M., Gascoyne R.D., Rosen S.T., Tobinai K. (2011). Peripheral T-cell lymphoma. Blood.

[42] Swerdlow S.H., Campo E., Pileri S.A., Harris N.L., Stein H., Siebert R., Advani R., Ghielmini M., Salles G.A., Zelenetz A.D. (2016). The 2016 revision of the World Health Organization classification of lymphoid neoplasms. Blood.

[43] Hill L.C., Rouce R.H., Wu M.J., Wang T., Ma R., Zhang H., Mehta B., Lapteva N., Mei Z., Smith T.S. (2024). Antitumor efficacy and safety of unedited autologous CD5.CAR T cells in relapsed/refractory mature T-cell lymphomas. Blood.

[44] Pan J., Tan Y., Shan L., Deng B., Ling Z., Song W., Feng X., Hu G. (2022). Phase I study of donor-derived CD5 CAR T cells in patients with relapsed or refractory T-cell acute lymphoblastic leukemia. JCO.

[45] Alotaibi F.M., Min W.P., Koropatnick J. (2024). CD5 blockade, a novel immune checkpoint inhibitor, enhances T cell anti-tumour immunity and delays tumour growth in mice harbouring poorly immunogenic 4T1 breast tumour homografts. Front. Immunol..

[46] Dai Z., Mu W., Zhao Y., Cheng J., Lin H., Ouyang K., Jia X., Liu J., Wei Q., Wang M. (2022). T cells expressing CD5/CD7 bispecific chimeric antigen receptors with fully human heavy-chain-only domains mitigate tumor antigen escape. Signal Transduct. Target. Ther..

[47] Schwarz S., Linnebacher M. (2023). CD5: From antiquated T cell marker to immunotherapy’s new hope. Sig. Transduct. Target Ther..

[48] Patel R.P., Ghilardi G., Zhang Y., Chiang Y.-H., Xie W., Guruprasad P., Kim K.H., Chun I., Angelos M.G., Pajarillo R. (2024). CD5 deletion enhances the antitumor activity of adoptive T cell therapies. Sci. Immunol..

[49] AbouYabis A.N., Shenoy P.J., Flowers C., Lechowicz Mary J. (2007). Response and Survival Rates in Patients with Peripheral T-Cell Lymphoma Treated with Anthracycline-Based Regimens: A Comprehensive Meta-Analysis. Blood.

[50] Chen K.H., Wada M., Pinz K.G., Liu H., Lin K.W., Jares A., Firor A.E., Shuai X., Salman H., Golightly M. (2017). Preclinical targeting of aggressive T-cell malignancies using anti-CD5 chimeric antigen receptor. Leukemia.

