# Supplementary material for: Prevalence and Prognostication of CD5+ Mature T-Cell Lymphomas
Source: Cancers (Basel). 2024 Oct 9;16(19):3430. doi: 10.3390/cancers16193430 (PMC11476060; doi:10.3390/cancers16193430)
Supplement: Supplementary file 1 [file cancers-16-03430-s001.zip › cancers-3184935-supplementary.pdf]

Supplemental Table S1: Multivariable modeling of diagnostic variables on CD5 status

Variable	Estimate	Std. Error	t value	Pr(>|t|)
(Intercept)	1	3.59E-15	2.79E+14	0.00
Subtype: PTCL-NOS	-2.30E-15	9.23E-16	-2.49	0.01 *
Subtype: TFH TCL / AITL	-2.18E-15	9.22E-16	-2.36	0.02 *
ECOG > 2	1.28E-15	6.59E-16	1.94	0.06
subtype: ATLL	-2.38E-15	1.44E-15	-1.65	0.10
B-symptoms +	-9.08E-16	5.88E-16	-1.54	0.13
Subtype: ALK+ ALCL	-2.13E-15	1.49E-15	-1.43	0.16
Subtype: ENKTCL	-1.91E-15	1.53E-15	-1.25	0.21
Subtype: EATL	-3.17E-15	2.92E-15	-1.08	0.28
Sex	4.59E-16	5.45E-16	0.84	0.40
Subtype: BIA-ALCL	-2.55E-15	3.19E-15	-0.80	0.43
Subtype: SPTCL	-1.69E-15	2.44E-15	-0.69	0.49
Subtype: CD30+ CTCL/LPD	-2.18E-15	3.16E-15	-0.69	0.49
Subtype: PCGD-TCL	-1.11E-15	2.45E-15	-0.45	0.65
Age of Diagnosis	-8.62E-18	2.06E-17	-0.42	0.68
B-symptoms -	-5.94E-16	1.51E-15	-0.39	0.70
Subtype: T cell PLL	-1.07E-15	3.18E-15	-0.34	0.74
Stage: 1	-7.83E-16	2.66E-15	-0.29	0.77
Stage: 2	-3.42E-16	1.40E-15	-0.24	0.81
EN. Disease +	7.02E-16	3.02E-15	0.23	0.82
ECOG <2	3.85E-16	1.83E-15	0.21	0.83
EN Disease -	6.24E-16	2.98E-15	0.21	0.84
Stage: 4	2.44E-16	1.39E-15	0.18	0.86
Stage: 3	1.72E-17	1.37E-15	0.01	0.99

* p-value <= 0.05, have statistical significance
